# Antimicrobial activity, phytochemical screening of crude extracts, and essential oils constituents of two *Pulicaria spp.* growing in Sudan

**DOI:** 10.1038/s41598-020-74262-y

**Published:** 2020-10-13

**Authors:** Ebtihal Alsadig Ahmed Mohamed, Ali Mahmoud Muddathir, Magda Abker Osman

**Affiliations:** 1 Department of Horticulture, Faculty of Agriculture, University of Khartoum, Shambat, 13314 Khartoum, Sudan; 2 Horticultural Crops Research Center, Agricultural Research Corporation, Shambat 30, Khartoum, Sudan; 3 Medicinal and Aromatic Plants and Traditional Medicine Research Institute, National Centre for Research, 2404 Khartoum, Sudan

**Keywords:** Microbiology, Plant sciences

## Abstract

The search for plant extracts with highly antimicrobial activity has been increased nowadays. This study evaluated the antimicrobial activity of *Pulicaria crispa* (Forsk.) Oliv., and *Pulicaria undulata* (L.) C.A.Mey., which have been used traditionally in Sudan as insect replants. The antimicrobial activity was evaluated against six pathogenic microorganisms, four bacteria (two Gram-positive; *Bacillus subtilis* and *Staphylococcus aureus*, two Gram-negative; *Escherichia coli*, and *Pseudomonas aeruginosa*), and two fungi (*Aspergillus niger* and *Candida albicans*) using disc diffusion method. The extraction of the crude extracts was done by maceration. The essential oils were extracted by hydro-distillation. Phytochemical screening was done using reference method. Essential oils were analyzed using Gas Chromatography Mass Spectrometry. The results indicated that all used the microorganisms were sensitive to the plants extracts. Results of the preliminary phytochemical screening showed the presence of saponins, comarins, tannins, sterols, and triterpenes, and absence of alkaloids, anthraquinones, and flavonoids. Twenty-eight and forty-five constituents were identified in *P. crispa* and *P. undulata,* essential oils, respectively. The main constituents in the essential oil of *P. crispa* were 1,4-ditert butylbenzene (22.81%), caryophyllene (13.19%), carvone (11.80%), and neryl(s)-2-methylbutanoate (10.33%), and for *P. undulata* were camphor (44.48%), and thymyl acetate (10.31%). Data from this study could be used for developing of natural bioactive agents to improve human health.

## Introduction

The search for substances with highly antimicrobial activity has been one of the most intensive field of research to minimize the risk of infectious diseases that caused by bacteria, fungi, viruses, and parasites, which are pathogenic to humans. Plants extracts are still the major sources of many therapeutic agents including antimicrobial agents for the treatment of infectious diseases^1,2^.

The family Asteraceae includes about 100 genera, and 2300 species. The genus *Pulicaria* is one of these genera, and it includes 100 species distributed worldwide^3^. Seven species of the genus *Pulicaria* have been reported in Sudan, namely; *P. attenuata, P. crispa, P. dysenterica var. stenophylla, P. grantii*, *P. petiolaris, P. undulata,* and *P. vulgaris*^4^. Only three species of the genus *Pulicaria* have been found in Khartoum State, which are *P. crispa*, *P. grantii,* and *P. undulata*^5^.

*Pulicaria crispa* (Forsk.) Oliv. (synonym *Francoeuria* (Forsk.)) and *Pulicaria undulata* (L.) C.A.Mey., are two wild aromatic plants growing in Sudan. Their local names are "alrabul", and "altager", and these plants contain plenty of compounds with medicinal importance^6^. *P. crispa* and *P. undulata* are annual herbs or sometimes perennial sub-shrubs, with small yellow flowers containing essential oil characterized by a strong aromatic odor. These plants are one of the most widespread desert plants growing wild in Sudan, Saudi Arabia, Kuwait, Iran, Iraq, Southern Egypt, Afghanistan, Pakistan, India, and parts of north & west tropical Africa^7–9^.

Different *Pulicaria* species have been traditionally used in several countries to repel insects, to treat back pain, to treat intestinal disorders, to treat inflammation, and to reduce influenza, and common cold symptoms. *Pulicaria* species contain many bioactive compounds such as monoterpenes, sesquiterpene acetylenes, flavonoids, isocomene, alkaloids, glycosides, comarins, and tannins^1,6^.

Many studies have reported that, *P. undulata* have been used traditionally in Sudan against alopecia, as a tea substitute, as an antispasmoic, as an ingredient of local perfumes. In addition, the plant has been used in folk medicine in many countries as an antiepileptic, as galactagogue, and as insect repellent (farmers are used to but it inside the vegetables packing containers). Also it has been reported that the essential oil of *P. undulata* has been used in the preparation of tonics, as sedative, and as an antibacterial agent^9–13^.

A group of researcher reported that *P. crispa* was found to have many folkloric medicinal uses in many countries. It has been used for many years in conventional medicine for the cure of heart diseases due to its antioxidative nature, also it has been used by the people of Sudan, Southern Egypt and Saudi Arabia to treat inflammation, as an antimicrobial agent, as an insect repellent, for the treatment of colds, coughs, colic, excessive sweating, and as carminative^9,14,15^.

The antimicrobial activity of *P. crispa* and *P. undulata,* methanolic crude extracts and essential oils were studied against pathogenic bacteria, and fungi. A preliminary phytochemical screening of the crude extracts, and the constituents of the essential oils have also been investigated in the present study.

## Results and discussion

### The yield percentages of the crude methanolic extracts and the essential oils

Data in Table 1 show the yield percentages of the crude methanolic extracts, and the essential oils. The crude methanolic extraction yield of *P. crispa* and *P. undulata* was 22.6% and 23%, respectively, (w/w) on dry weights bases. The essential oils of both species obtained by hydro-distillation from whole plants of *P. crispa* and *P. undulata* was 0.1% and 0.4%, respectively, (v/w) on dry weights bases. The odor of the aerial parts (stems, leaves, and flowers) of *P. undulata* was sharper than that of *P. crispa,* due to the percentages, and essential oils constituents of the two species^14^. Reported that the methanolic crude extract of *P. crispa* yielded 27% crude extract^16^. Stated that the essential oil extracted from *P. crispa* grown wild in Egypt reach to 0.23%. These differences in yield percentages of crude extract and essential oils could by refer to the differences in environmental condition.

**Table 1:** The yield percentages of the crude methanolic extracts and essential oils of *P. crispa and P. undulata.*

Plant name	Yield (%)	
	Crude methanolic extract (%)	Essential oil (%)
*P. crispa*	22.6	0.1
*P. undulata*	23.0	0.4

### Antimicrobial activity of the crude methanolic extracts and the essential oils of *P.crispa* and *P.* *undulata*

The extracts of the studied plants exhibited varying degrees of inhibition activity against the tested bacteria (Table 2); and the results were expressed in terms of the diameter of the growth-inhibition zone (clear zones). The results clearly showed that tested bacteria were susceptible to the four extracts. There were significant differences (*p* < 0.05) in mean diameter inhibition zone between the four extracts. *P. crispa* methanolic crude extract showed high activity against *S. aureus* (19 mm), and moderate activity against *B. subtilis* (16 mm), *E. coli* (15 mm), and *P. aeruginosa* (15 mm). *P. undulata* methanolic crude extract showed high activity against *B. subtilis* (18 mm), and moderate activity against *E. coli* (16 mm), *P. aeruginosa* (16 mm), and *S. aureus* (15 mm), which is also similar in activity to Tetracycline. Regarding the essential oil of *P. crispa,* it shows high activity against *S. aureus* (18 mm), which is higher than positive controls; also it showed moderate activity against *B. subtilis* (17 mm), *P. aeruginosa* (17 mm), and *E. coli* (16 mm). *P. crispa* essential oil showed high activity against *S. aureus* (18 mm). Regarding the essential oil of *P. undulata*, it showed high activity against *B. subtilis* (25 mm), *P. aeruginosa* (24 mm), and was found to be moderately active against *E. coli* (17 mm) and *S. aureus* (17 mm), even though it is higher in activity than tetracycline (16 mm). Generally the essential oil of *P. undulata* showed high activity against *B. subtilis*, and *P. aeruginosa* compared to positive controls.

**Table 2:** Antibacterial activity of *P. crispa* and *P. undulata* methanolic crude extracts, and the essential oils against the bacterial microorganisms.

Extracts	Bacterial microorganisms mean diameter inhibition zone (mm)			
	*B. subtilis*	*E. coli*	*P. aeruginosa*	*S. aureus*
*P. crispa* methanolic crude extract	16^b^	15^b^	15^b^	19^a^
*P. undulata* methanolic crude extract	18^b^	16^b^	16^b^	15^b^
*P. crispa* essential oil	17^b^	16^b^	17^b^	18^ab^
*P. undulata* essential oil	25^a^	17^b^	24^a^	17^ab^
Gentamicin*	19^b^	22^a^	17^b^	16^ab^
Tetracycline*	23^a^	16^b^	16^b^	15^b^
0.05% LSD	3.727	3.246	3.702	3.324

(*)Positive control at the concentration of (40 mg/ml).

Means separated by least significant difference (LSD) test at *p* < 0.05.

Means followed by a similar letter(s) in the same column are not significantly different at *p ≤ *0.05 according to the least significant difference test.

Antifungal activity of the methanolic crude extracts, and the essential oils of *P. crispa* and *P. undulata* was presented in Table 3. There were significant differences (*p* < 0.05) in mean diameter inhibition zone between the four extracts. All the extracts showed activity against the two fungal microorganisms. However, *P. undulata* essential oil showed the highest activity against the two fungi compared to the other extracts. Generally the extracts showed high activity against *C. albicans; P. undulata* essential oil 23 mm, *P. undulata* methanolic crude extract 21 mm, *P. crispa* methanolic crude extract 20 mm, and *P. crispa* essential oil 19 mm, While the extracts were found to be relatively less active against *A. niger*; *P. undulata* essential oil 22 mm, *P. crispa* essential oil 21 mm, *P. undulata* methanolic crude extract 20 mm, and *P. crispa* methanolic crude extract 19 mm, which was lower than positive controls (Nystatin (26 mm) and Clotrimazole (34 mm).

**Table 3:** Antifungal activity of *P. crispa* and *P. undulata* methanolic crude extracts, and the essential oils against the fungal microorganisms.

Extracts	Fungal microorganisms mean diameter inhibition zone (mm)	
	*Aspergillus niger*	*Candida albicans*
*P. crispa* methanolic crude extract	19^c^	20^ab^
*P. undulata* methanolic crude extract	20^c^	21^ab^
*P. crispa* essential oil	21^c^	19^b^
*P. undulata* essential oil	22^c^	23^ab^
Clotrimazole*	34^a^	24^a^
Nystatin*	26^b^	14^c^
0.05% LSD	3.978	4.172

(*) Positive control. Means separated by least significant difference (LSD) test at *p* < 0.05.

Means followed by a similar letter(s) in the same column are not significantly different at *p ≤ *0.05 according to the least significant difference test.

### Minimum inhibitory concentrations (MIC) of *P.crispa* and *P.undulata* methanolic crude extracts, and essential oils

The results of MIC presented in Table 4 showed that all microorganisms were very susceptible to the minimum inhibitory concentration of *P. crispa* essential oil (6.25 mg/ml) except for *B. subtilis* the MIC value was 25 mg/ml, similarly all microorganisms were susceptible to minimum inhibitory concentration of *P. undulata* essential oil (6.25 mg/ml), except for *E. coli* and *B. subtilis* with MIC value (50 mg/ml). Regarding *P. crispa* methanolic crude extract the minimum inhibitory concentration was 6.25 mg/ml for *P. aeruginosa* and *A. niger,* 25 mg/ml for *E. coli* and *S. aureus,* 50 mg/ml for *C. albicans* and 100 mg/ml for *B. subtilis.* While, minimum inhibitory concentration of *P. undulata* methanolic crude extract was 6.25 mg/ml for *A. niger* and *C. albicans,* 12.5 mg/ml for *P. aeruginosa* and *S. aureus,* the MIC was 50 mg/ml for *E. coli* and 100 mg/ml for *B. subtilis.*

**Table 4:** Antimicrobial activity expressed as minimum inhibitory concentration (MIC; mg/ml) of the methanolic crude extracts and essential oils from *P. crispa* and *P. undulata.*

Extracts	*A. niger*	*B. subtilis*	*C. albicans*	*E. coli*	*P. aeruginosa*	*S. aureus*
*P. crispa* methanolic crude extract	6.25	100	50	25	6.25	25
*P. undulata* methanolic crude extract	6.25	100	6.25	50	12.5	12.5
*P. crispa* essential oil	6.25	25	6.25	6.25	6.25	6.25
*P. undulata* essential oil	6.25	50	6.25	50	6.25	6.25

### Preliminary phytochemical screening of the crude extracts of* P.crispa* and *P.undulata*

Data presented in Table 5 show the preliminary phytochemical examination of the methanolic crude extracts of *P. crispa* and *P. undulata*, which were rich in sterols, and terpenes, tannins, comarins, saponins. At the same time data confirm the absence of alkaloids, flavonoids, and anthraquinones. These results are in line with the findings of previous researches^16–20^. These groups of phytochemicals might be responsible for the observed antimicrobial activity *P. crispa* and *P. undulata*.

**Table 5:** Preliminary phytochemical screening of the methanolic crude extracts of *P. crispa* and *P. undulata*.

Secondry metabolites	*P. crispa*	*P. undulata*
Alkaloids	−	−
Anthraquinones	−	−
Comarins	+ ++	+ +
Flavonoids	−	−
Saponins	++	+ ++
Sterols	++	+ ++
Tannins	+ +	+ ++
Triterpenes	++	+ ++

+Trace, ++Moderate, +++High, −Absent.

### The chemical constituents of *P. crispa* and *P. undulata* essential oils

The hydro-distillation of the dry aerial parts of *P. crispa,* and *P. undulata* grown in Sudan gave yellow- colored essential oils. The percentage composition, and identification of each *Pulicaria* species essential oil are listed in Tables 6 and 7.

**Table 6:** The chemical composition of the essential oil of *P. crispa*.

Peak no	Compound	Retention time	%	Formula
1	Beta-Myrcene	12.296	0.18	C_10_H_16_
2	Limonene	13.485	2.74	C_10_H_16_
3	Eucalyptol	13.580	0.72	C_10_H_18_O
4	Linalool	15.845	0.09	C_10_H_18_O
5	Levomenthol	18.110	0.49	C_10_H_20_O
6	Dihydrocarvyl acetate	18.796	0.56	C_12_H_10_O_2_
7	Carvone	20.201	11.80	C_10_H_14_O
8	Isopulegol acetate	22.480	0.51	C_12_H_20_O_2_
9	Cis- carvyl acetate	23.418	0.21	C_12_H_18_O_2_
10	Neryl (S)-2-methylbutanoate	23.809	0.61	C_15_H_26_O_2_
11	11,Methylene-Tricyclo	24.037	0.38	C_13_H_20_
12	Lavandulyl acetate	24.912	7.36	C_12_H_20_O_2_
13	Caryophyllene	24.959	13.19	C_15_H_24_
14	Beta-ocimene	25.835	0.69	C_10_H_16_
15	Neryl (S)-2-methylbutanoate	27.070	10.33	C_15_H_26_O_2_
16	Butanoic acid	27.100	4.52	C_4_H_8_O_2_
17	1-(3-Isobutyryl-bicyclo[1.1.1]pent-1-yl)-2-methylpropan-1-one	27.220	0.30	C_13_H_20_O_2_
18	4-Hexadecen-6-yne, (Z)-	28.298	0.61	C_16_H_28_
19	2(1H)-Naphthalenone, 4a,5,8,8a-tetrahydro-1,1,4a-trimethyl	28.861	0.60	C_13_H_18_O
20	1,4-ditert-butylbenzene	29.051	22.81	C_14_H_22_
21	Limonen-6-ol, pivalate	29.284	1.70	C_15_H_24_O_2_
22	Camphore	29.664	0.94	C_10_H_16_O
23	Longipinocarveol, trans	30.300	2.16	C_15_H_24_O
24	Gamma-muurolene	30.360	11.72	C_15_H_24_
25	Alpha.-Cadinol	30.684	0.81	C_15_H_26_O
26	1H-3a,7-Methanoazulene, octahydro-1,4,9,9-tetramethyl	30.767	1.03	C_15_H_26_
27	10–12-Pentacosadiynoic acid	31.076	2.42	C_25_H_42_O_2_
28	Benzenepropanoic acid, 3,7-dimethylocta-2,6-dienyl ester	38.194	0.52	C_19_H_26_O_2_
Total			100.00

**Table 7:** Chemical composition of the essential oil of *P. undulata*.

Peak no	Compound	Retention time	%	Formula
1	2-Hexenal	8.199	0.28	C_6_H_10_O
2	Bicyclo	11.893	3.46	C_9_ H_17_ N
3	Linalool oxide	15.500	1.64	C_10_ H_18_ O_2_
4	Trans linalool oxide	16.002	0.67	C_10_ H_18_ O_2_
5	Linalool	16.419	1.10	C_10_H_18_O
6	Nonanal	16.419	1.18	C_9_ H_18_ O
7	Ho-trienol	16.475	0.07	C_12_H_18_O_2_
8	Isobornyl formate	16.571	0.31	C_11_H_18_O_2_
9	2-Cyclohexen-1-ol	19.077	2.90	C_6_H_10_O
10	heptan-2-ol	19.917	1.92	C_7_H_16_O
11	2-Cyclohexen-1-one	20.656	0.52	C_6_H_8_O
12	Camphor	21.013	44.48	C_10_H_16_O
13	Cyclohexanone	21.219	1.48	C_10_H_16_O_2_
14	2-(1-methyl-2-oxopropyl	21.645	0.78	C_11_H_16_N_2_O_3_S
15	Thymol	22.074	1.69	C_10_H_14_O
16	Cis-jasmone	22.646	0.96	C_11_ H_16_ O
17	Thymohydroquinone	25.006	2.45	C_12_ H_18_ O_2_
18	Thymyl acetate	25.571	10.31	C_12_ H_16_ O_2_
19	Propanoic acid	27.082	1.77	C_14_H_24_O_2_
20	Humulen	27.142	0.28	C_15_H_24_
21	Geranyl propionate	27.187	0.13	C_13_ H_22_ O_2_
22	Geranyl propionate	27.615	0.25	C_13_ H_22_ O_2_
23	Delta.-cadinene	27.649	0.04	C_15_ H_24_
24	Cyclohexanemethanol	28.119	1.43	C_17_H_28_O_2_
25	Geranyl propionate	28.812	0.55	C_13_ H_22_ O_2_
26	Phenol, 3-methyl-5-(1-methylethyl)	29.225	0.65	C_12_ H_17_ N O_2_
27	Butanoic acid	29.294	0.14	C_15_H_26_O_2_
28	Isopropyl dimethyl chlorosilane	29.390	0.37	C_5_H_13_
29	Caryophyllene oxide	29.471	0.44	C_15_ H_24_ O
30	Tau.-Cadinol	29.659	1.43	C_15_H_26_O
31	Alpha.-Cadinol	31.002	2.79	C_15_H_26_O
32	Juniper camphor	31.324	2.76	C_15_ H_26_ O
33	Ethanone	31.464	0.94	C_10_H_12_O_4_
34	Azulenol	31.743	3.40	C_15_ H_26_ O
35	1-Hexen	32.188	1.03	C_14_H_20_O
36	1-(4-Isopropylphenyl)	36.592	0.18	C_16_H_24_O
37	Hexadecanoic acid	36.686	0.22	C_16_ H_32_ O_2_
38	1-Ppenylpentan	37.612	1.68	C_11_ H_14_ O
39	Propanoic acid	38.862	0.73	C_16_H_20_O_5_
40	9,12-Octadecadienoic acid	40.462	0.17	C_18_H_32_O_2_
41	Oleic acid	40.817	0.29	C_18_H_34_O_2_
42	Hexacosane	40.895	0.37	C_26_ H_54_
43	Hexatriacontane	46.815	0.21	C_36_H_74_
44	Borane	50.466	0.13	C_5_ H_13_ B
45	Oxirane	57.667	1.39	C_18_H_36_O
Total			100.00	

GC–MS analysis of the essential oils resulted in identification of twenty-eight constituents in *P. crispa* essential oil, and forty-five constituents in *P. undulata* essential oil. The main constituents of the essential oil of *P. crispa* were 1,4-ditert-butylbenzene (22.81%), caryophyllene (13.19%), carvone (11.80%), neryl (s)- 2-methylbutanoate (10.33%). In addition, the main constituents of the essential oil of *P. undulata* were camphor (44.48%), thymyl acetate (10.31%), bicycle (3.46%), and azulenol (3.40%), other minor constituents have been identified in the essential oils of *P.* *crispa* and *P.* *undulata*. Both linalool, and camphor are presented in the essential oil of *P. crispa* and *P. undulata*. Result of *P. undulata* essential oil constituents agrees with those obtained by^21^ of *P. undulata* collected from Yemen in linalool, camphor, and thymol.

The bactericidal properties of *P. undulata* essential oil were due to the presence of thymol, and thymol derivatives, which were found to have a significant antimicrobial activity^22^.

## Conclusions

This study showed that the essential oils and the methanolic crude extracts of *P. crispa* and *P. undulata,* inhibited the growth of various tested species of Gram-positive, Gram-negative bacteria, and fungi. Generally, we can conclude that *P. crispa* and *P. undulata* methanolic crude extracts, and essential oils have antimicrobial activity. 3- caryophyllene oxide and carvomenthenone were the major compounds in *P. crispa* and *P. undulata* essential oil, respectivily. The above-mentioned results may provide a promising topic for further in vitro and in vivo studies to develop curative plant extracts from *P. crispa* and *P. undulata.*

## Methods

This study was carried out at the Medicinal and Aromatic Plants and Traditional Medicine Research Institute, National Center for Research, Khartoum, Sudan.

### Plant material

Plants were selected randomly followed by antimicrobial assays. Aerial parts of *P. crispa* and *P.* *undulata* were collected during the flowering stage in July 2015 from different locations in Khartoum State, Sudan. The plants materials were taxonomically authenticated at the herbarium of Medicinal and Aromatic Plants and Traditional Medicine Research Institute, *P. crispa* voucher specimen number is W-1995-41-MAPTRI-H, and *P. undulata* voucher specimen number is A-1995-126-MAPTRI-H.

### Preparation of the plant materials

*P. crispa* and *P. undulata,* plants parts were freed from dust, and foreign material, then dried indoors at room temperature for three days, powdered, then kept in plastic containers at room temperature until used.

### Preparation of the crude extracts

*P. crispa* and *P. undulata,* methanolic crude extracts were prepared by maceration of the dried powdered plants materials in organic solvent (methanol). Twenty grams of each plant sample were extracted using 50 ml of absolute methanol as solvent. The mixture was allowed to stand for 72 h at room temperature with daily filtration using a standard filter paper (Whatman No. 2, England). The solvent was evaporated under reduced pressure to dryness using rotary evaporator, then the crude extracts have left to dry at room temperature for three days. The yield percentages were determined by dividing the weight of extract by the weight of the sample multiplied by 100. The extracts were stored at 4 °C until used^23^.

### Preparation of the essential oils

*P. crispa* and *P. undulata* essential oils were prepared by hydro-distillation of the dried powdered plants materials in water. 100 grams of each sample were submitted to hydro-distillation for four hours using Clevenger- type apparatus (Duran West Germany). The obtained essential oils were calculated as a relative’s percentage (v/w), and dried over anhydrous sodium sulfate, filtered, and stored at 4 °C until used^16^_._

### Antimicrobial activity screening of the methanolic crude extracts, and the essential oils of *P.crispa* and *P.undulata,* microorganisms

The antimicrobial activity of *P. crispa* and *P. undulata* methanolic crude extracts, and the essential oils were evaluated by disc diffuison method using ATCC (American Type Culture Collection), and NCTC (National Collection of Type Cultures) strains. The strains were four bacterial strains; two Gram-positive (*Bacillus subtilis* (NCTC 8236) and *Staphylococcus aureus* (ATCC 25923)), two Gram-negative (*Escherichia coli* (ATCC 25922), and *Pseudomonas aeruginosa* (ATCC 27853)), and two fungi (*Aspergillus niger* (ATCC9763) and *Candida albicans* (ATCC7596)).

### Preparation of the microorganism culture

All the test microorganisms were inoculated on blood agar, and nutrient agar plates. The bacterial strains were incubated at 37 ºC for 24 h, and the fungal strains were incubated at 37 °C for 48 h in the inverted position, incubated aerobically, and the obtained growth were then stored in the refrigerator at 4 °C till used.

### Determination of antimicrobial activity of the methanolic crude extracts, and the essential oils of *P.crispa*, and *P.undulata,* by disc diffuison method

The paper disc diffusion method was used to screen the antimicrobial activity of the plants extracts, and performed by using Mueller Hinton agar (MHA). The experiment was carried out according to the National Committee for Clinical Laboratory Standards^24^. The bacterial suspension was diluted with a sterile physiological solution to 108 CFU/ml. 100 µl of the bacterial suspension were swabbed uniformly on the surface of MHA and the inoculum was allowed to dry for five minutes. The sterilized filter paper discs (Whatman No. 2, England) were placed individually on the surface of the MHA and impregnated with 20 µl of samples solution. The inoculated plates with bacteria were incubated at 37 °C for 24 h in the inverted position and 48 h for the fungal strains. The diameters (mm) of the inhibition zones were measured; the values of the antimicrobial activity were expressed as the mean of inhibition zones (mm) with three replicates for each treatment. Gentamicin, Tetracycline, Clotrimazole and Nystatin served as positive controls. The results of the diameters of the zones of inhibitions of the extracts were interpreted as sensitive; (> 18 mm), intermediate (14–17 mm), and resistant (< 14 mm)^17,18^.

### Determination of the minimum inhibitory concentration (MIC)

The principle of the agar plate dilution is the inhibition of growth on the surface of the agar by the plant extracts incorporated into the medium was used to determine the minimum inhibitory concentration (MIC) according to^25^. The MICs of the extracts were used in concentrations (6.25–100 mg/ml). Agars were prepared in the series of increasing concentrations of the plant extract. The bottom of each plate was marked off into four segments. The organisms tested were growing in broth overnight to contain 108 CFU/ml. Loop-full of diluted culture is spotted with a standard loop that delivers 0.001 ml on the surface of the segment and incubation for 24 h or 48 h at 37 °C. The MIC were determined visually in the agar as the lowest concentrations of the extracts in which no bacterial/fungal growth was visible. The MICs values were express as mg/ml.

### Phytochemical screening of the methanolic crude extracts

The methanolic crude extracts were subjected to qualitative examination for the detection of various phytochemical constituents (saponins, comarins, alkaloids, anthraquinones, tannins, flavonoids, sterols, and triterpenes) using standard procedures^26–28^.

### Essential oils analysis by the gas chromatography-mass spectrometry (GC–MS)

*P. crispa* and *P. undulata,* essential oils were analyzed using Shimadzu Gas Chromatography Mass Spectrometry Apparatus (Japan) (GC.MS- QP2010 Ultra). Analysis was carried out on a Varian 3400 system equipped with a DB-5 fused silica column (30 m length × 0.25 mm diameter, 0.25 μm film thickness). Helium was used as the carrier gas (1.2 ml/min), and the program used was four minutes isothermal at 35 °C , following by 40–240 °C at the rate of 4 °C/min, then held at 260 °C, for three minutes, the injection temperature was 250 °C. The components of the essential oils were identified by library searches^29^, based on comparing their retention indices, and mass spectra with those obtained from authentic samples, and/or the NIST/NBS, Wiley libraries, and the literature.

### Statistical analysis

The collected data were subjected to the analysis of variance (ANOVA), and the means were separated using the least significant difference (LSD) at *p ≤ * 0.05 and at *p ≤ * 0.01.
